# Effect of Mo_2_C Addition on the Tribological Behavior of Ti(C,N)-Based Cermets

**DOI:** 10.3390/ma16165645

**Published:** 2023-08-16

**Authors:** Hao Qiu, Xiaoqiang Li, Cunliang Pan, Jiafeng Fan

**Affiliations:** National Engineering Research Centre of Near-Net-Shape Forming Technology for Metallic Materials, South China University of Technology, Guangzhou 510640, China; hao.qiu@rwth-aachen.de (H.Q.); 201910100488@mail.scut.edu.cn (C.P.); fjf201909@163.com (J.F.)

**Keywords:** Ti(C,N)-based cermets, hot press sintering, Mo_2_C, fracture toughness, tribological properties

## Abstract

Due to the excellent properties of Ti (C,N)-based ceramics, such as high hardness, excellent wear resistance, exceptional thermal deformation resistance, and sound chemical stability, they have been widely used in cutting tools or molds. Thus, revealing their tribological behavior against hard materials is of great significance. Some studies have reported the tribological behavior of Ti(C,N)-based cermets and hard cermets, but so far, the effects of Mo_2_C additions on the frictional properties of Ti(C,N)-based cermets are still unclear. In this study, Ti(C,N)-10WC-1Cr_3_C_2_-5Co-10Ni-x Mo_2_C cermets (x = 4, 6, 8, 10 and 12 wt.%) were sintered using a vacuum hot-pressing furnace. Furthermore, the core–rim morphologies of the sintered samples were observed in SEM images. Then, the wear resistance of the cermets was studied against a Si_3_N_4_ ball at a 50 N load using the fretting wear test. Finally, the wear mechanism was characterized using a combination of SEM, EDS and XPS. The experimental results indicated that the wear mechanisms of the cermets were mainly abrasive wear, adhesive wear, and the formation of an oxide film. As the content of Mo_2_C increased from 4 wt.% to 12 wt.%, the friction coefficient and wear volume had a variation law of first decreasing and then decreasing, and reached minimum values at 6 wt.% and 12 wt.%, and the lowest friction coefficient and wear rate were 0.49 and 0.9 × 10^−6^ mm^3^/Nm, respectively. The 6 wt.% Mo_2_C greatly improved the hardness and fracture toughness of the cermet, while the 12 wt.% Mo_2_C promoted the formation of an oxide film and protected the friction surface. The cermet with 6 wt.% Mo_2_C is recommended because it has comprehensive advantages in terms of its mechanical properties, tribological properties, and cost.

## 1. Introduction

Ti(C,N)-based cermets exhibit significant potential for diverse applications, including cutting tools, sea ling rings, and wear parts. This is owing to their remarkable attributes, such as high hardness, exceptional wear resistance, extraordinary resistance to thermal deformation, and sound chemical stability [1,2,3,4,5]. Ti(C,N)-based cermets have demonstrated superior oxidation resistance and high temperature toughness when compared to traditional WC–Co cemented carbide. As a result, they have become an increasingly competitive rival [6,7]. Consequently, Ti(C,N)-based cermets have found widespread and successful implementation within the metal cutting industry, especially in high-speed cutting applications [8,9].

Ti(C,N)-based cermets comprise two phases: the cermet phase, such as Ti(C,N), which has high hardness, and a metal binder phase, such as Co and Ni, which is used as a binder phase to bond with the cermet phase to make Ti(C,N)-based composites with a high relative density, high hardness, and high toughness [10]. However, the poor wettability between the binder phase and the Ti(C,N) hard phase, as well as the low fracture toughness of the cermets, hinder the application of Ti(C,N) cermets.

So far, many technologies have been developed to fabricate Ti(C,N)-based cermets, such as hot-press sintering (HPS) [11], microwave sintering [12], and hot isostatic pressing sintering (HIPS) [13]. Microwave sintering can improve the density of the cermets, but causes a reduction in the hardness to a certain extent. HIPS does not need a mold, but the preparation efficiency is low. Compared with microwave sintering and HIPS, HPS can economically obtain highly dense materials, a uniform fine-grained microstructure, and excellent physical and mechanical properties with high reliability [14]. Thus, HPS has been widely used for sintering and developing various materials, including composite materials such as hard metals and cermets [15,16].

In the Ti(C,N)-based cermets, Mo_2_C is generally used as a sintering aid to enhance the wettability between the Ti(C,N) grain and the binder phase, and to improve the fracture toughness of cermets [17]. Earlier studies have indicated that the addition of Mo_2_C to Ti(C,N)-based cermets is beneficial to the wettability between the Ti(C,N) cermet phase and the (Ni, Co) metallic phase, and a finer microstructure and better mechanical properties have been obtained [2,18,19,20,21]. 

TiCN-based cermets have been thoroughly studied for their wear behavior under a wide range of dry ambient testing conditions. Extensive investigations have been carried out to understand the friction and wear characteristics of TiCN-based cermets when interacting with steel. Previous researchers have reported a considerable variation in wear rates, varying from 10^−7^ mm^3^/Nm to 10^−6^ mm^3^/Nm. The complicated wear behavior of TiCN-based cermets is defined by adhesion, abrasion, tribo-chemical wear, oxidation, plastic deformation, and fracture according to the experimental conditions of wear testing [22,23,24,25]. 

There are significant differences in the tribological performance of different friction pairs; however, there is little information on hard cermet counter bodies, and the effects of Mo_2_C additions on the frictional properties of Ti(C,N)-based cermets against hard cermet counter bodies are still unclear. The relationship between the addition of Mo_2_C, the “core–rim” structure and the strengthening and toughening mechanism remains unclear among the studies. As the application of TiCN cermet is extending to hard metals and ceramics, in order to evaluate their full potential, a thorough grasp of tribological wear behavior against hard counter bodies is very necessary. The aim of this study is to clarify the effects of Mo_2_C on the microstructure and tribological properties of Ti(C,N)-based cermets. 

## 2. Materials and Methods

### 2.1. Preparation of Cermets

The particle sizes and purity of the initial powders are listed in Table 1. The powders were precisely weighed and mixed based on the specified composition provided in Table 2. The content range of Mo_2_C addition was determined based on the previous studies of our team and other researchers [18,26,27]. Subsequently, they were wet-milled in an ethanol bath using YG8 cemented carbide balls in a planetary ball mill (QM-3SP4, NanDa Instrument Plant, Nanjing, China) for 24 h at a rotational speed of 200 r/min. The powder/ball/ethanol mass ratio was maintained at 1:5:0.8. Finally, the mixture was dried at 70 °C in an electrically heated drying oven. 

Before the sintering, the mixed powder was filled into a cylindrical graphite mold that was 30 mm in diameter and 60 mm in height. A layer of graphite paper was placed between the graphite mold and the powder. Under vacuum conditions (10^−3^ Pa), the powders were first pre-pressed at room temperature and then hot-pressing sintered at 1500 °C and 20 MPa for 1 h, following furnace cooling. 

**Figure 1:**
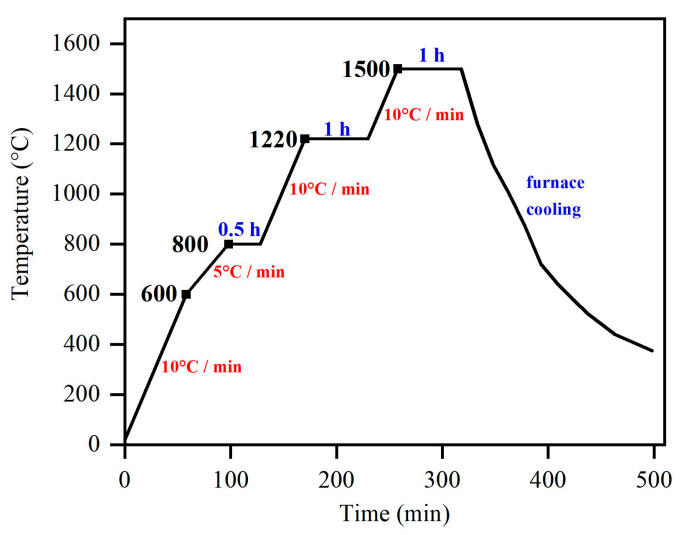
The temperature curve of the vacuum hot press sintering of TiCN-based cermets.

Figure 1 shows the sintering curve of the Ti(C,N)-based cermets. In order to achieve a more uniform furnace temperature and facilitate the release of gases (CO_2_, CO, N_2_, etc.) before the liquid-phase sintering stage (in which the binder phases Ni and Co melted), specific dwell times of 30 min at 800 °C and 60 min at 1220 °C were implemented. These conditions were chosen to promote the densification of the sample [28,29]. 

### 2.2. Characterization

After sintering, the specimens were cut into 8 × 8 × 5 mm^3^ pieces using an EDM wire-cutting machine. Subsequently, the pieces were ground and polished for metallographic analysis. The surface morphology of the sample was examined using a metallographic microscope (Leica DMi8 C) and a high-resolution scanning electron microscope (FEI Nova Nano SEM 430). The densities of the samples were determined using the Archimedes immersion method.

The Vickers hardness was measured using a THVP-30 hardness tester (Beijing Era United Technology Co., Ltd., Beijing, China) with a 10 kgf load, and the obtained result was determined according to the average of seven measurements. To determine the fracture toughness (KIC) of the specimens, the crack lengths departing from the Vickers indentation corners were measured, and the expression derived by Shetty et al. was employed [30]:

(1) $$K_{IC}=A\sqrt{\frac{HV\cdot P}{\sum_{i=1}^{4}l_{i}}}$$

where *HV* is the hardness (N·m^−1^); *P* is the load (N); *l* is the sum of crack lengths (mm); and *A* is a constant, which is determined by the following equation: *A* = 1/[3(1 − ν^2^)(2^1/2^π^5/2^ tan θ)^1/3^], where ν is Poisson’s ratio of the material and θ is the angle of the opposite faces of Vickers indenter (2θ = 136° in our test). For ν = 0.22 (typical value of cermet), *A* is calculated as 0.0889 [31]. In our study, the hardness of specimens was tested under a load of 98 N. Thus, the Equation can be simplified as follows:

(2) $$K_{IC}=0.88\sqrt{\frac{{HV}_{10}}{\sum_{i=1}^{4}l_{i}}}$$

where *K_IC_* is the fracture toughness (MPa·m^1/2^); *HV*_10_ is the Vickers hardness (GPa); and *l_i_* is the length of four cracks at the corner of the indentation (mm).

### 2.3. Fretting Wear Test

The fretting wear test was conducted utilizing an oscillating friction and wear tester (SRV IV, Optimol, Munich, Germany), with a ball-on-block contact configuration. The detailed parameters of the fretting wear tests are shown in Table 3. The specimens were cut into blocks with dimensions of 8 × 8 × 8 mm for the test, and their testing surfaces were polished. Si_3_N_4_ balls with a diameter of 10 mm, a hardness of 1700 HV, and a surface roughness of 0.025 µm were chosen as the counterbody due to their high hardness and chemical inertness under wear conditions. The experiment was conducted at room temperature (approximately 25 °C) with an air humidity of 40–45%, and the coefficient of friction (CoF) was continually recorded during the test. A load of 5 N was preloaded for 5 min before the test for running-in. In order to ensure that enough friction products could be observed, previous work was studied [32,33,34], and a high load of 50 N was set. The mean CoF and average wear volume of each material state were determined using the results of at least three conducted tests. 

Both before and after the fretting wear test, the specimen was cleaned in ethanol and then dried. The profiles of the wear scars were measured using a universal 3D profilemeter (UP Dual Model, Rtec Instruments, San Jose, CA, USA), and the wear volume of the specimens was calculated using the Gwyddion software (Version 2.61, Czech Metrology Institute, Brno, Czechia). 

In addition, the wear surfaces of the samples were characterized using X-ray photoelectron spectroscopy (XPS, AXIS SUPRA+, Shimadzu, Kyoto, Japan). The C1s peak was used as a fixed reference point at 284.8 eV to calibrate all binding energies. Thermo Avantage software was employed for the analysis of the obtained spectra (version 5.9922). 

## 3. Results and Discussion

### 3.1. Phase Analysis of TiCN-Based Cermets

**Figure 2:**
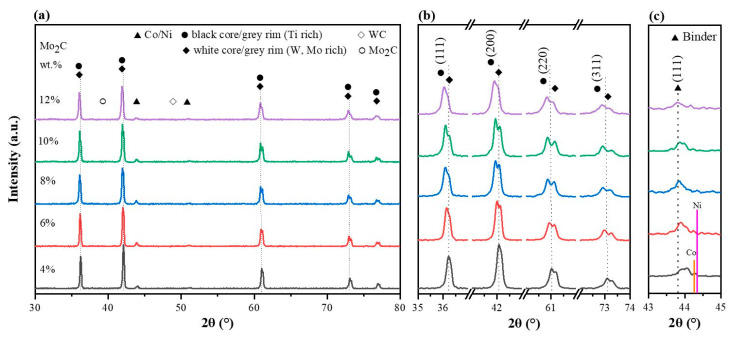
XRD patterns of sintered cermets with various Mo_2_C contents: (**a**) the complete pattern; (**b**) enlarged peaks of hard phases; (**c**) enlarged peaks of binder and pure Co/Ni.

The XRD analysis was carried out to confirm the crystallographic structures of the TiCN-based cermets, as shown in Figure 2. The hard phase (Ti,M)(C,N) can be observed in all specimens, where M represents W or/and Mo in the molecular formula (Ti,M)(C,N). The binder-phase Ni–Co–M peaks, which are slightly adrift from the pattern of Ni and Co, indicate the existence of Ti, Mo or W dissolved in the binder phase. No diffraction peaks corresponding to WC or Mo_2_C were detected in any of the specimens. This suggests that most and even all of the WC and Mo_2_C underwent dissolution and formed hard phases of (Ti,W,Mo)(C,N) solid solution. 

The lattice constants calculated based on the XRD patterns of various phases are shown in Table 4. When the Mo_2_C content exceeds 8 wt.%, the lattice constants of the white core and grey rim exhibit minimal variation. Consequently, the content of W and Mo remains stable in the white core–grey rim, aligning with the findings of Lindahl et al.’s research [35]. 

### 3.2. Core–Rim Morphology of Sintered TiCN-Based Cermet

**Figure 3:**
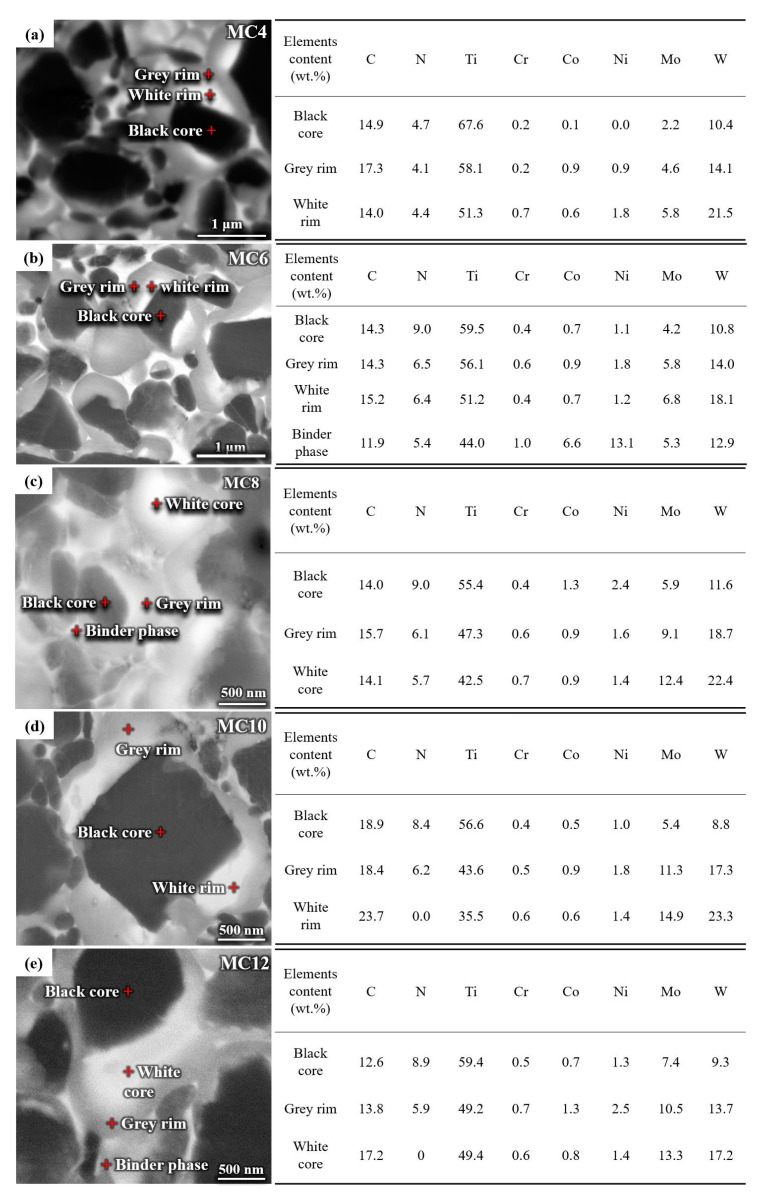
SEM–BSE micrographs and EDS point analysis of the specimens with various Mo_2_C contents: (**a**) 4 wt.%, (**b**) 6 wt.%, (**c**) 8 wt.%, (**d**) 10 wt.%, (**e**) 12 wt.%.

Figure 3 shows the microstructure of the Ti(C,N)-based cermets with Mo_2_C contents of 4 wt.%, 6 wt.%, 8 wt.%, 10 wt.% and 12 wt.%. Due to the addition of Mo_2_C, the Ti(C,N)-based cermets form a unique core–rim structure, namely black core–white inner rim–gray outer rim structure. As shown in Figure 3a–e, elements such as C, N, Ti, Cr, Co, Ni, Mo, and W were detected in the black core phase and rim phase of the five types of cermets. The composition difference between the black core phase and the rim phase is manifested in the higher content of C, N, and Ti in the black core phase, while it is manifested in the higher content of W and Mo in the rim phase, which is related to the formation of the black core phase and the rim phase. As the sintering temperature increases, Mo, TiC, TiN, and Ni diffuse with each other. Before the liquid phase appears, the undissolved TiC and TiN hard-phase particles become black core phases. Then, during the solid-state sintering stage, due to the occurrence of dissolution and precipitation, a white inner rim phase rich in W and Mo is formed around the black core phase. And in the later stage of liquid-phase sintering, excess TiC, TiN, WC, and Mo_2_C precipitate from the bonding phase, forming a gray outer rim phase around the white inner rim phase. As seen in Figure 3, when the content of Mo_2_C increases from 4 wt.% to 6 wt.%, the microstructures are significantly uniform, and the hard-phase grains are significantly refined, and the average grain diameter decreases from 1 μm to 0.5 μm. This is because sufficient Mo_2_C can form a complete ring phase via the dissolution precipitation mechanism, thereby improving the wettability of the bonding relative to the hard phase and reducing the direct contact between hard grains. However, as the Mo_2_C content continues to increase to 12 wt.%, the volume fraction of the rim phase increases and the proportion of core phase decreases. In addition, as the Mo_2_C content increases, the Mo content in the black core phase, white inner rim phase, and gray outer rim phase increases, while the Ti content in the gray outer rim phase decreases, which is also related to the increase in the volume fraction of the rim phase.

### 3.3. Mechanical Properties of Sintered TiCN-Based Cermet

**Figure 4:**
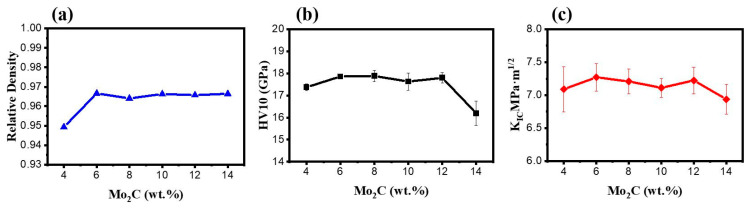
Mechanical properties of cermets: (**a**) relative density, (**b**) hardness and (**c**) fracture toughness.

The correlation between the Mo_2_C content and the relative density of the Ti(C,N)-based cermets is presented in Figure 4. As can be observed, the cermet achieves a constant relative density of more than 96% when the content of Mo_2_C is greater than 6 wt.%. This is due to the fact that a higher Mo_2_C concentration improves the wettability between the hard phase and the metal phase, and enhances the sinterability of cermet, leading to a denser microstructure [27]. As can be observed from Figure 4b,c, Mo_2_C also has a significant effect on the mechanical characteristics of cermets. The addition of 6 weight percent of Mo_2_C results in optimal mechanical properties, with a Vickers hardness of 17.86 GPa and a fracture toughness of 7.27 MPa.m^1/2^, which are significantly higher than the Ti(C,N)-based cermets without Mo_2_C addition (16.34 GPa [32] and 7.0 MPa·m^1/2^ [36]). The strengthening effects were mainly due to the core–rim structure induced by the addition of Mo_2_C.

As the Mo_2_C content is increased from 4 wt.% to 6 wt.%, the fracture toughness shows improvement, attributed to the refinement of hard-phase grains and the presence of thin rims. Thin rims are advantageous for fracture toughness, because thick rims can be more easily passed through by cracks or induce them under loading [37,38]. When the Mo_2_C content exceeds 6 wt.%, excessive Mo_2_C leads to the agglomerations of fine Ti(C,N) particles, which reduce the wettability of the binder phase on the cermet phase [39]. The excessive Mo_2_C addition inhibits the development of rims surrounding large Ti(C,N) cores as well. These cause negative effects on the fracture toughness of cermet. Additionally, the high fracture toughness of cermets also depends on the mean free path of the large binder [40], together with the increasing W content in the rims [41]. Therefore, the fracture toughness of cermets decreases with the increase in the Mo_2_C content, which results in a lower W content in the rims and a smaller binder mean free path.

### 3.4. Frictional Behavior and Wear Results

**Figure 5:**
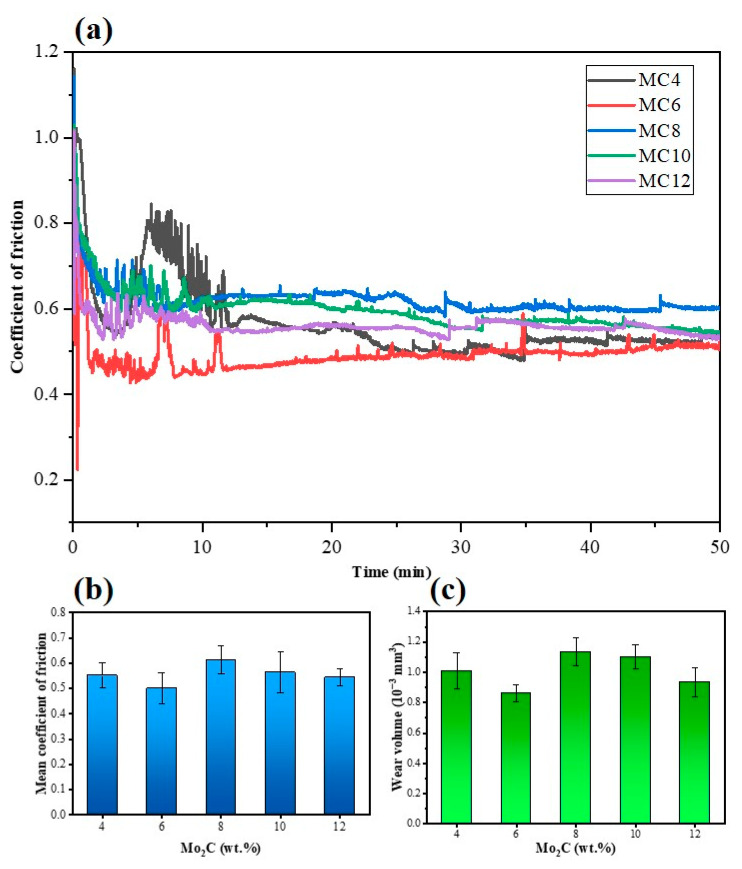
Wear results of Ti(C,N)-based cermets: (**a**) dynamic CoF; (**b**) mean CoF; (**c**) wear volume.

**Figure 6:**
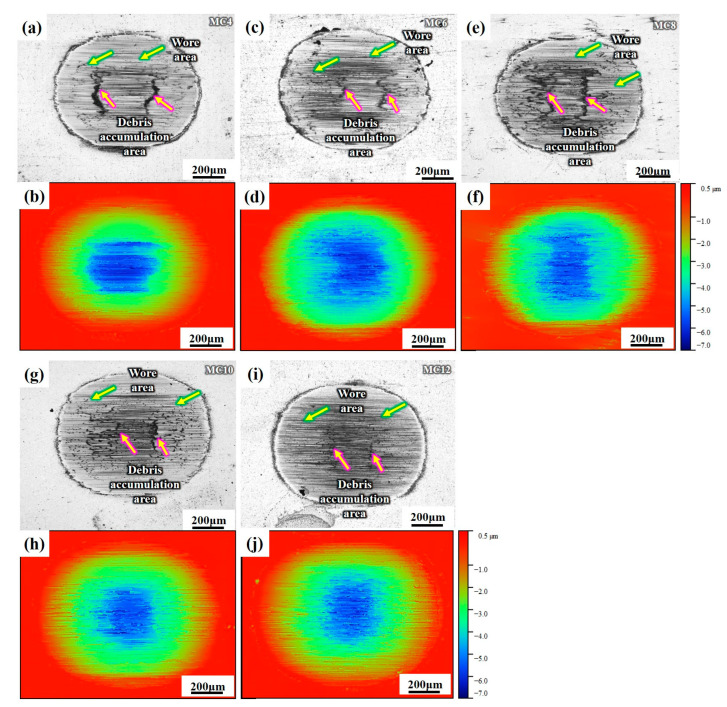
Images of the wear tracks on the Ti(C,N)-based cermets with various Mo_2_C contents: (**a**,**b**) 4 wt.%, (**c**,**d**) 6 wt.%, (**e**,**f**) 8 wt.%, (**g**,**h**) 10 wt.%, (**i**,**j**) 12 wt.%.

The tribological properties of Ti(C,N)-based cermets were assessed. Figure 5 shows the dynamic coefficients of friction (CoF), the mean coefficients of friction, and the wear volume of Ti(C,N)-based cermets with different Mo_2_C contents. MC14 is excluded due to its poor mechanical properties. As shown in Figure 6, three sorts of areas—unworn areas, debris accumulation areas, and worn areas—were formed on the attack surface of the cermets after the wear experiment along the sliding direction. Following the fretting wear test, the morphology of the worn surfaces of the cermets was investigated.

As demonstrated in Figure 5a, CoFs of all specimens show clear fluctuations throughout the running-in phase (10–20 min after the beginning), which is mostly brought on by the direct contact between rough peaks and the friction surfaces. After that, in the stable stage of the CoF curves, the mean CoFs are determined and the wear volume is estimated. As seen in Figure 5 and Figure 6, when the content of Mo_2_C increases from 4 wt.% to 12 wt.%, the friction coefficient and wear volume have a variation law of first decreasing, then increasing, and then decreasing. Specifically, as the Mo_2_C content increases from 4 wt.% to 6 wt.%, the wear volume (Figure 5c) and the mean CoF (Figure 5b) decrease due to an increase in the relative density hardness of the specimens (Figure 4b) When the Mo_2_C content goes from 6 wt.% to 8 wt.%, the hardness of the specimen diminishes, and the results of the friction test show that the CoF climbs to 0.61 and the wear volume also increases. Interestingly, as the Mo_2_C content rises from 8 wt.% to 12 wt.%, the CoF again declines from 0.61 to 0.54, and the wear loss drops as well. When the Mo_2_C contents are 6 wt.% and 12 wt.%, respectively, the specimens show low CoFs and wear volumes. Even so, the wear loss of the cermets with 12 wt.% Mo_2_C is slightly lower.

### 3.5. SEM and EDS Analysis of Worn Surfaces

**Figure 7:**
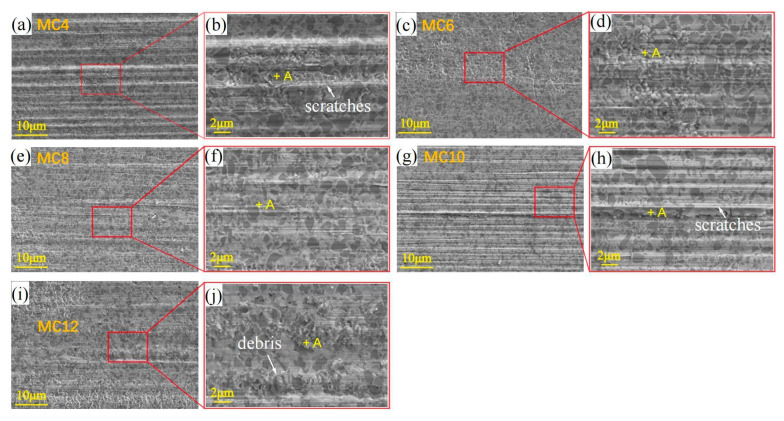
SEM–BSE micrographs of the cermets’ worn surfaces after fretting at a 50 N load with various Mo_2_C contents: (**a**,**b**) 4 wt.%, (**c**,**d**) 6 wt.%, (**e**,**f**) 8 wt.%, (**g**,**h**) 10 wt.%, (**i**,**j**) 12 wt.%.

After the fretting wear test, the detailed morphology of the worn surfaces of the cermets was studied in order to establish the relationship between the Mo_2_C content and the characteristics of the tribological surface, as shown in Figure 7. As seen in Figure 7a,b, when the Mo_2_C content is 4 wt.%, some scratches can be observed on the surface of the cermet, and Si is not detected, which means that the wear between the friction pairs is mainly abrasive wear. When the Mo_2_C content is low, the formed rim phase is incomplete, and the rim phase cannot completely wrap the hard phase, which results in the hard phase having a larger grain size and a poor interfacial bonding strength between the rim phase and the hard phase. Therefore, during friction and wear, the rim phase in the cermet is broken and the hard phase becomes abrasive particles.

As seen in Figure 7c,d, when the Mo_2_C content is 6 wt.%, the surface of the cermet has fewer scratches and debris, and the surface is smooth, which is mainly because the cermet with 6 wt.% Mo_2_C has the best mechanical properties (hardness and fracture toughness, as shown in Figure 4); in addition, the proper core–rim structure can improve the wettability between the liquid bonding phase and the solid hard phase, as well as the uniformity of the organizational structure, so as to increase the interfacial bonding strength between the rim phase and the hard phase. In the process of friction and wear, the rim phase is not easily broken, so it has good friction performance.

When the Mo_2_C content increases to 8–10 wt.%, as can be seen in Figure 7e–h, more scratches and debris appear on the surface of the cermet. From Table 5, there is a small amount of Si on the surface of the cermet after friction, which indicates that there exists abrasive wear and adhesive wear. With the increase in the Mo_2_C content, the thickness of the rim phase further increases, and the uniformity of the structure deteriorates. At this time, the brittleness of the rim phase plays a major role, the solid solution strengthening effect of the rim phase is not obvious, and the interfacial bonding strength between the rim phase and the hard phase decreases. During the friction and wear process, the rim phase is prone to breakage, resulting in abrasive wear and adhesive wear. However, as can be seen in Figure 7i,j, when the Mo_2_C content increases to 12 wt.%, there are fewer scratches and debris on the surface of the cermet, and the surface is smoother. According to the experimental results shown in Figure 7, the wear resistance of metal cermets with a Mo_2_C content of 6 wt.% and 12 wt.% is better, which is consistent with their wear volume in Figure 5c. 

From Table 5, as the Mo_2_C content increases, the content of oxygen on the surface of the cermet after friction and wear increases, while Si mainly appears on the surface with a Mo_2_C content of 10 wt.%. This indicates that oxide forms during the friction and wear process, and Figure 7f shows that when the Mo_2_C content is 12 wt.%, the cermet surface wear is reduced. The formation of an oxide film improves the wear performance of the cermet and avoids further wear on the surface of the friction pair.

### 3.6. XPS Analysis of Worn Surfaces

**Figure 8:**
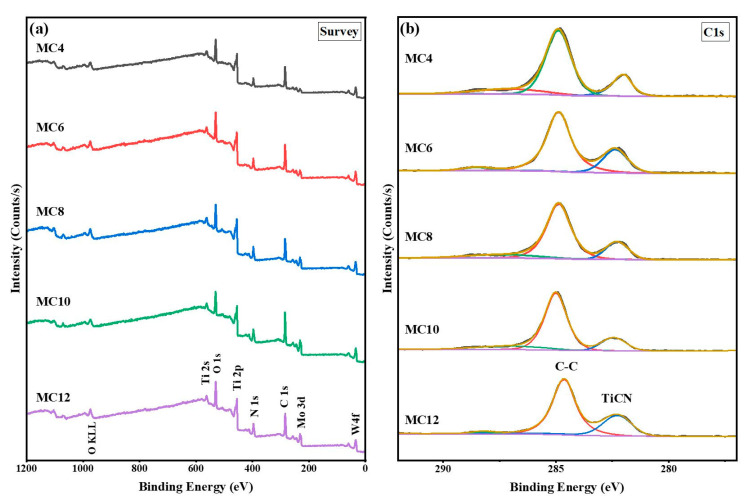
XPS survey spectra of specimens with various Mo_2_C contents: (**a**) wide scan; (**b**) C 1s.

XPS was used to analyze the surface characteristics of the sample. Figure 8a shows the wide-scan survey spectra of the cermets, corresponding to W4f, Mo3d, C1s, N1s, Ti2p and O1s at 231.9 eV, 284.8 eV, 396.4 eV, 455.44 eV and 530.37 eV, respectively. As can be seen from the spectra, the presence of O, Ti, Mo, C, and N was confirmed [42,43]. C1s at 284.8 eV was used as a standard for calibration [44], while C1s at 281.9 eV was assigned to TiCN in this study [45].

**Figure 9:**
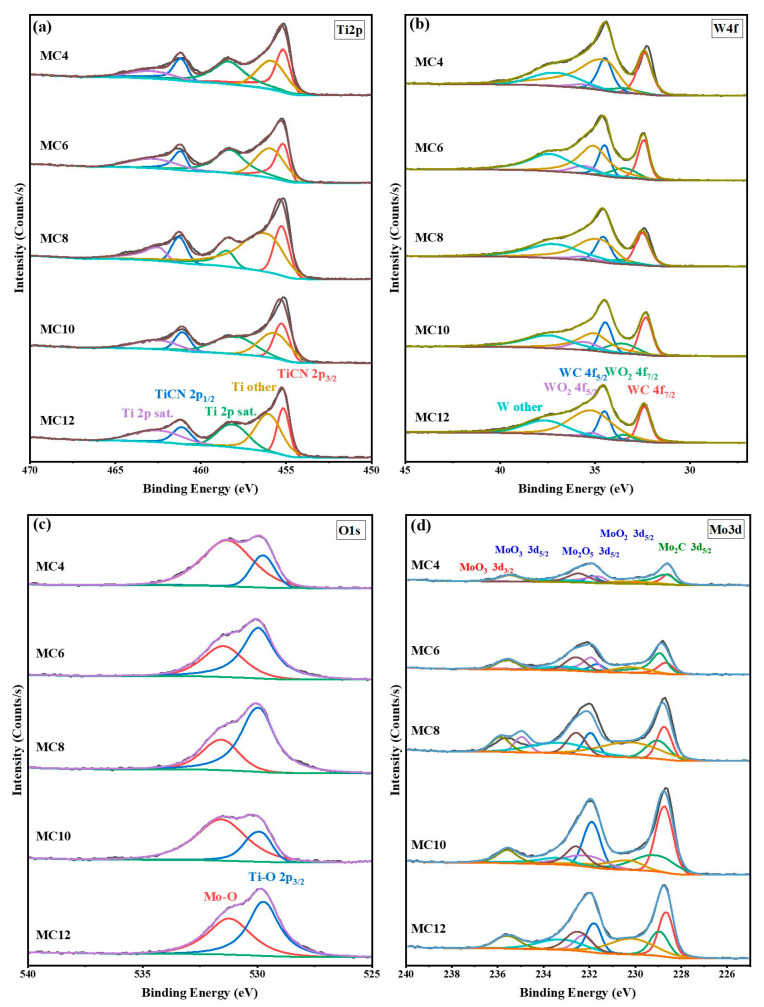
XPS spectra of specimens with various Mo_2_C contents: (**a**) Ti 2p; (**b**) W 4f; (**c**) O 1s; (**d**) Mo 3d.

As can be seen in Figure 9, the Ti2p spectra show that there are two spin splitting peaks, namely 3/2 and 1/2, and that the distance between the two spin splitting peaks is about 6.0 eV. The satellite peak of TiN is also detected [46]. The W4f spectra exhibit two distinct double peaks: one at 32.1 and 34.0 eV for W4f7/2, and another at 35.3 and 37.5 eV for the W4f5/2 spectra. The low-energy side of the W4f double peaks is thought to be connected to the W–N bonding [47]. The XPS spectra of the O1s energy region for the Ti(C,N)-based cermets include two peaks. That at 529.76 eV is deemed to be due to Mo or W oxides, and that at 531.32 eV is consistent with the Ti oxides [46,48]. 

The XPS narrow-scan spectrum of Mo 3d is shown in Figure 9d. Two peaks at 231.92 eV and 235.41 eV can be attributed to the presence of MoO_3_ formation in the tribological films [42], while that at 228.6 eV corresponds to Mo in the hard phase (Ti,W,Mo)CN. This indicates that friction triggers the transformation of Mo_2_C into Mo oxides. It is noteworthy that the presence of MoO_2_ and MoO_3_ on the friction interface enhances the wear resistance properties [49]. This accounts for the observed decrease in the CoF and wear volume with the increasing Mo_2_C content.

Table 6 shows the relative atomic content of each compound calculated from the XPS spectrum fitting results. According to Table 6, it can be found that the products on the friction surface include TiN, WC, WO_2_, Mo_2_C, MoO_2_, Mo_2_O_5_, MoO_3_ and other compounds. When the Mo_2_C content increases from 4 wt.% to 12 wt.%, the relative contents of TiN, WC and WO_2_ show a variation law of first increasing, then decreasing, and then increasing. When the content of Mo_2_C is 6 wt.% and 12 wt.%, the content of WC has the maximum value (17.7 wt.% and 30.5 wt.%, respectively). The increase in WC helps to improve the toughness of the cermets, and thus enhances their friction and wear properties. In addition, as can be seen in Table 6, when the content of Mo_2_C increases from 4 wt.% to 12 wt.%, the content of MoO_3_ presents a variation law of first decreasing and then increasing, and the generation of MoO_3_ plays a certain protective role on the friction surface. When the content of Mo_2_C is 4 wt.%, the content of MoO_3_ is 26.9 wt.%. However, the friction coefficient and wear performance are poor (Figure 5 and Figure 7), which indicates that the friction and wear behavior of the ceramics at this time is mainly determined by the mechanical properties (Figure 4). The adhesive phase and hard phase are prone to shape deformation, resulting in particle stripping along the wear track, and the resulting abrasive wear increases the wear amount and friction coefficient. When the content of Mo_2_C is 6 wt.% and 8 wt.%, and the content of MoO_3_ is 24.1 wt.% and 19.3 wt.%, respectively, the mechanical properties of the cermet are close to each other (Figure 4). However, Mo_2_C with a content of 8 wt.% has the largest friction coefficient and wear amount (Figure 5 and Figure 7), which indicates that a sufficiently thick oxide friction film can avoid the further wear of the friction pair. When the content of Mo_2_C is 12 wt.%, the content of MoO_3_ is 20.5 wt.%, and the mechanical properties of the cermet are better (Figure 5); in addition, the tribological properties of the cermet are better under the dual action of mechanical properties and friction film. According to the results shown in Figure 6, Figure 7, Figure 8 and Figure 9 and Table 6, it can be inferred that the friction and wear behavior of the TiCN cermet is determined by the mechanical properties and oxide friction film. 

The effects of Mo_2_C addition on the wear resistance of cermets could be divided into several aspects. It was confirmed that the addition of Mo_2_C could increase the hardness. Moreover, compared with Ti(C,N)-based cermets without Mo_2_C [50], the addition of Mo_2_C also enhanced the ability to form oxide film during the wear process, which greatly helped in the protection of the cermets’ surface from tear. However, the excessive addition of Mo_2_C could cause a decrease in the toughness, which was also illustrated by other researchers [27]. Considering the comprehensive influence of Mo_2_C, cermets with 6 wt.% Mo_2_C hold the best tribological properties.

## 4. Conclusions

Ti(C,N)-based cermets have been widely used in cutting tools or molds, and thus revealing their tribological behavior against hard materials is of great significance. This article conducted fretting wear tests and studied the effect of Mo_2_C on the tribological behavior of Ti(C,N)-based cermets. The main conclusions are as follows:

1. The addition of Mo_2_C could induce the formation of a “core–rim” structure, which exhibited significant strengthening effects on the mechanical properties, including hardness and fracture toughness. The core phase is mainly composed of Ti(C,N), while the rim phase is mainly composed of (W,Mo,Ti)(C,N). When the content of Mo_2_C is 6 wt.%, the cermets have optimal mechanical properties.
2. Due to the Mo_2_C addition and “core–rim” structure, the wear mechanisms of cermets are mainly abrasive wear, adhesive wear, and oxidation wear. As the content of Mo_2_C increases from 4 wt.% to 12 wt.%, the friction coefficient and wear volume have a variation law of first decreasing, then increasing, and then decreasing, and reach minimum values at 6 wt.% and 12 wt.%, which is the result of the comprehensive effect of the Mo_2_C strengthening and the oxide film.
3. The tribological behavior of Ti(C,N)-based cermets was determined according to the hardness, toughness and formation of an oxide film. On the one hand, the addition of Mo_2_C helped to increase the hardness and to form an oxide film, which plays a role in protecting the surface. On the other hand, the excessive carbide reduced the toughness and increased the risk of crushing. Combining these findings with practical engineering applications, and considering the mechanical properties, tribological properties, and cost factors, cermets with a Mo_2_C content of 6 wt.% are feasible.

## Figures and Tables

**Table 1:** The particle sizes and the purity of the initial powders.

Powder	TiCN	WC	Mo_2_C	Cr_3_C_2_	Ni	Co
Particle size (μm)	<1	<0.8	2–5	<0.8	<5	<5
purity	99.9%	99.9%	99.9%	99.9%	99.8%	99.8%

**Table 2:** The nominal composition of Ti(C,N)-based cermets (wt.%).

Specimen	Composition (wt.%)					
	TiCN	Co	Ni	Mo_2_C	WC	Cr_3_C_2_
MC4	Bal.	5	10	4	10	1
MC6	Bal.	5	10	6	10	1
MC8	Bal.	5	10	8	10	1
MC10	Bal.	5	10	10	10	1
MC12	Bal.	5	10	12	10	1
MC14	Bal.	5	10	14	10	1

**Table 3:** Parameters of fretting wear test.

Parameter	Set Value
Stroke (μm)	200
Frequency (Hz)	20
Preloading Time (min)	5
Preloading Force (N)	5
Normal Force (N)	50
Loading Time (min)	50

**Table 4:** Lattice parameters (Å) of various phases based on the XRD patterns in Figure 2.

Samples	Mo_2_C (wt.%)	Black Core/Grey Rim	White Core/Grey Rim	Binder
MC4	4	4.287	-	3.557
MC6	6	4.296	4.286	3.568
MC8	8	4.302	4.290	3.572
MC10	10	4.302	4.289	3.573
MC12	12	4.308	4.290	3.577

**Table 5:** EDS analysis of point A in Figure 7 on the worn surfaces of cermets after fretting (wt.%).

Samples	C	N	O	Ti	Si	Cr	Co	Ni	Mo	W
MC4	10.03	9.87	1.36	66.73	-			0.53	2.68	8.79
MC6	9.05	9.66	3.83	61.4	-		1.12	2.15	3.79	9.01
MC8	8.02		16.63	50.24	-	0.45	0.7	1.19	9.75	13.01
MC10	4.23	0	15.92	49.7	1.19	0.52	0.38	0.7	12.17	15.2
MC12	5.24	1.26	14.76	52.82	-	0.5	2.19	4.45	9.01	9.79

**Table 6:** Analysis of XPS spectra on the worn surface of cermets.

	Elements and Compounds	Binding Energy (eV)	at. %	Elements and Compounds	Binding Energy (eV)	at. %
MC4	Ti 2p_3/2_	455.16	7.51	Mo_2_C 3d_5/2_	228.56	11.69
	Ti 2p_1/2_	461.16	8.31	Mo_2_C 3d_3/2_	231.86	11.66
	Ti 2p_3/2_ sat.	458.35	36.57	MoO_2_ 3d_5/2_	228.61	17.93
	Ti 2p_1/2_ sat.	462.93	13.37	MoO_2_ 3d_3/2_	231.61	17.74
	Ti others	455.84	34.25	Mo_2_O_5_ 3d_5/2_	230.17	7.01
	WC 4f_7/2_	32.40	4.33	Mo_2_O_5_ 3d_3/2_	233.17	7.02
	WC 4f_5/2_	34.47	4.33	MoO_3_ 3d_5/2_	232.47	13.47
	WO_2_ 4f_7/2_	33.46	1.14	MoO_3_ 3d_3/2_	235.47	13.48
	WO_2_ 4f_5/2_	35.61	1.15			
	W 4f others	34.50	61.98			
	W 4f others	36.99	27.06			
MC6	Ti 2p_3/2_	455.16	5.31	Mo_2_C 3d_5/2_	228.64	7.91
	Ti 2p_1/2_	461.16	5.91	Mo_2_C 3d_3/2_	231.64	7.92
	Ti 2p_3/2_ sat.	458.26	35.15	MoO_2_ 3d_5/2_	228.93	22.16
	Ti 2p_1/2_ sat.	462.77	18.50	MoO_2_ 3d_3/2_	231.93	21.90
	Ti others	455.92	35.13	Mo_2_O_5_ 3d_5/2_	230.17	8.03
	WC 4f_7/2_	32.40	6.70	Mo_2_O_5_ 3d_3/2_	233.17	8.04
	WC 4f_5/2_	34.47	10.99	MoO_3_ 3d_5/2_	235.58	12.03
	WO_2_ 4f_7/2_	33.46	2.96	MoO_3_ 3d_3/2_	232.58	12.02
	WO_2_ 4f_5/2_	35.53	7.89			
	W 4f others	35.09	45.03			
	W 4f others	37.35	26.44			
MC8	Ti 2p_3/2_	455.16	6.70	Mo_2_C 3d_5/2_	228.64	11.44
	Ti 2p_1/2_	461.16	7.45	Mo_2_C 3d_3/2_	231.85	11.45
	Ti 2p_3/2_ sat.	458.36	3.58	MoO_2_ 3d_5/2_	228.93	5.91
	Ti 2p_1/2_ sat.	462.35	7.93	MoO_2_ 3d_3/2_	234.85	7.92
	Ti others	456.04	74.34	Mo_2_O_5_ 3d_5/2_	230.17	21.97
	WC 4f_7/2_	32.40	3.97	Mo_2_O_5_ 3d_3/2_	233.17	22.00
	WC 4f_5/2_	34.47	3.97	MoO_3_ 3d_5/2_	232.47	9.65
	WO_2_ 4f_7/2_	33.46	1.15	MoO_3_ 3d_3/2_	235.77	9.66
	WO_2_ 4f_5/2_	35.53	1.16			
	W 4f others	34.65	47.66			
	W 4f others	37.01	42.09			
MC10	Ti 2p_3/2_	455.16	9.30	Mo_2_C 3d_5/2_	228.64	21.04
	Ti 2p_1/2_	460.96	10.15	Mo_2_C 3d_3/2_	231.80	21.06
	Ti 2p_3/2_ sat.	457.81	12.06	MoO_2_ 3d_5/2_	228.93	13.24
	Ti 2p_1/2_ sat.	462.34	13.39	MoO_2_ 3d_3/2_	231.93	13.25
	Ti others	455.58	55.09	Mo_2_O_5_ 3d_5/2_	230.17	7.73
	WC 4f_7/2_	32.23	4.76	Mo_2_O_5_ 3d_3/2_	233.17	7.69
	WC 4f_5/2_	34.36	4.76	MoO_3_ 3d_5/2_	232.47	7.99
	WO_2_ 4f_7/2_	33.46	2.81	MoO_3_ 3d_3/2_	235.47	8.00
	WO_2_ 4f_5/2_	35.46	2.81			
	W 4f others	34.96	43.09			
	W 4f others	37.29	41.77			
MC12	Ti 2p_3/2_	455.16	8.98	Mo_2_C 3d_5/2_	228.64	17.07
	Ti 2p_1/2_	460.96	9.85	Mo_2_C 3d_3/2_	231.78	11.29
	Ti 2p_3/2_ sat.	458.10	21.60	MoO_2_ 3d_5/2_	228.93	11.69
	Ti 2p_1/2_ sat.	462.47	17.93	MoO_2_ 3d_3/2_	232.22	7.73
	Ti others	456.04	32.52	Mo_2_O_5_ 3d_5/2_	230.17	19.10
	WC 4f_7/2_	32.40	17.18	Mo_2_O_5_ 3d_3/2_	233.25	12.63
	WC 4f_5/2_	34.50	13.33	MoO_3_ 3d_5/2_	232.47	12.34
	WO_2_ 4f_7/2_	33.46	3.57	MoO_3_ 3d_3/2_	235.55	8.16
	WO_2_ 4f_5/2_	35.13	2.82			
	W 4f others	35.21	41.74			
	W 4f others	37.61	21.36			

## Data Availability

The data utilized in the present work can be obtained from this article.
